# Is physical inactivity and sedentary behavior associated with tumor stage in breast cancer patients? A cross-sectional study of Brazilian women

**DOI:** 10.31744/einstein_journal/2023AO0215

**Published:** 2023-06-06

**Authors:** Luana de Lima Queiroga, Rafael Mathias Pitta, Mayra de França Trevisani, Carla Giuliano de Sá Pinto Montenegro, Diogo Diniz Gomes Bugano, Aylton José Figueira, Julien Steven Baker, Danilo Sales Bocalini, Luciana Diniz Nagem Janot de Matos

**Affiliations:** 1 Hospital Israelita Albert Einstein São Paulo SP Brazil Hospital Israelita Albert Einstein, São Paulo, SP, Brazil.; 2 Universidade São Judas Tadeu São Paulo SP Brazil Universidade São Judas Tadeu, São Paulo, SP, Brazil.; 3 Hong Kong Baptist University Kowloon Tong China Hong Kong Baptist University, Kowloon Tong, China.; 4 Universidade Federal do Espírito Santo Vitória ES Brazil Universidade Federal do Espírito Santo, Vitória, ES, Brazil.

**Keywords:** Sedentary behavior, Exercise, Breast neoplasms, Neoplasm staging, Sitting position

## Abstract

**Objective:**

A comparative analysis of the association between sedentary behavior *versus* physical activity levels and tumor staging in women with breast cancer.

**Methods:**

The present research adopted a cross-sectional study design to recruit a total of 55 adult and elderly women newly diagnosed with breast cancer for data collection and analysis. Inclusion criteria involved patients in procession of a formal approval for participation in the study by the treating physician and those not hitherto subjected to the first cycle of chemotherapy.

**Results:**

Physical activity levels did not influence the pathological stage of breast cancer (p=0.26) or histological tumor grade (p=0.07) in the analyzed subjects. However, there was a significant association between physical activity levels and responsiveness to hormones (epidermal growth factor receptor (HER2), p<0.05) in the analyzed subjects. Significant difference was detected in the histological tumor grade in relation to the mean time spent sitting during the weekend (p<0.05). However, sedentary behavior had no influence on the tumor stage (p>0.05).

**Conclusion:**

Physical activity levels did not influence the tumor stage and histological tumor grade. Sedentary behavior had a significant influence on the histological tumor grade.

## Highlights

■ Physical activity level is associated with responsiveness to hormones in women with breast cancer.

■ Sedentary behavior is significantly related to the histological tumor grade in women with breast cancer.

**Figure:**
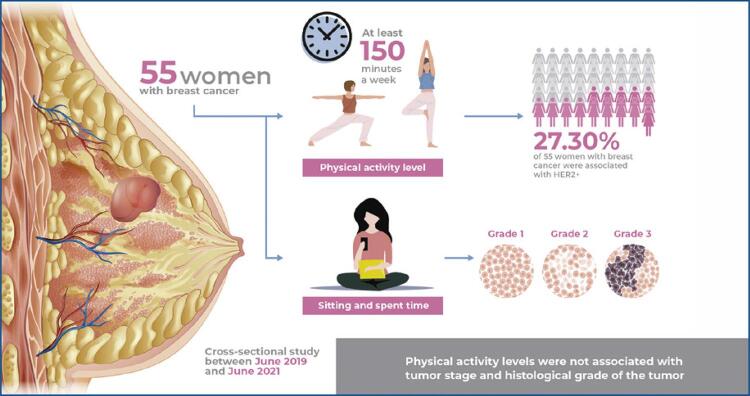

## INTRODUCTION

Breast cancer (BC) is the most common type of cancer in women,^(1)^ accounting for 2.1 million new annual cases worldwide. Breast cancer is associated with early morbidity, decreased health-related quality of life, and is the leading cause of cancer-related death among women globally.^(2)^

Breast cancer is a multifactorial heterogeneous disease characterized by abnormal proliferation of cells within the lobules and ducts of the breast.^(3)^ Diagnosis and classification of BC based on anatomopathological examination of the lesions is decisive for the formulation of treatment strategies. Staging of BC into five stages on a number scale of 0 through IV is determined by the tumor-node-metastasis (TNM) system centered on the guidelines of the American Joint Committee on Cancer (AJCC).^(4)^ The TNM system assigns the tumor stage based on the assessment of the anatomical extent of the disease, histological tumor grade, estrogen and progesterone receptor status, epidermal growth factor receptor (HER2), and the oncotype score.

Genetic factors and lifestyle are the major contributors to the etiology of BC.^(5)^ Obesity, excessive alcohol consumption, smoking, and physical inactivity are the dominant modifiable lifestyle factors that could be controlled or avoided to help prevent and manage BC. Sedentary behavior (SED) in occupational activities increases the risk of BC by 15.5%, regardless of the management of obesity, following a recent meta-analysis (31 studies including 13 prospective cohort and 18 case-control studies). Further, SED increases the risk of BC by 8% with an increment of 1% for every additional hour of sitting time as indicated by a meta-analysis that evaluated prospective data from 426,506 women. However, till date, only a small number of meta-analyses studies have aimed to analyze the relationship between menopause and BC risk.^(6)^

Physical activity levels (PAL) play an important role as a protective factor in the development of BC. The risk of BC is reduced by 14% in physically active women with active participation in leisure time, and during occupational, domestic, and transport activities as indicated by a recent meta-analysis that evaluated 35 prospective cohort studies, with data from 50,949,108 women.^(7)^ Further, depending on the magnitude of increase in PAL, the risk of recurrence and mortality is further decreased by 20-69% in women with BC.^(8)^

Although there exists several studies that investigated the role of PAL and SED in the onset and progression of BC using large prospective cohorts, only a limited number of studies have evaluated the correlation between these variables and the different stages of tumor in women diagnosed with BC. Thus, the role of PAL and SED in the classification of BC remains to be elucidated. Development of BC classifications were aimed at better understanding the specific clinical behavior of the disease, determination of prognosis, and formulation of appropriate treatment strategies, in addition to increasing the survival rate in women with BC.^(9)^ Therefore, deciphering the role of PAL and SED in the classification of BC would aid in the therapeutic intervention and development of novel treatment strategies in women diagnosed with BC.

## OBJECTIVE

A comparative analysis of the association between sedentary behavior versus physical activity levels and tumor staging in women with breast cancer.

## METHODS

### Design

This study used a cross-sectional design based on the data collected from women diagnosed with BC between June 2019 and 2021 at the *Hospital Municipal da Vila Santa Catarina Dr. Gilson de Cássia Marques de Carvalho; Hospital Israelita Albert Einstein* (PROADI-SUS - *Programa de Apoio ao Desenvolvimento Institucional do Sistema Único de Saúde*), São Paulo, Brazil.

The study was approved by the ethics committee of the *Hospital Israelita Albert Einstein* (CAAE: 92740718.3.0000.0071; # 2.993.056) and the *Secretaria Municipal da Saúde de São Paulo* (SMS/SP, CAAE: 92740718.3.3001.0086; # 3.090.567). All participants read and signed the informed consent form authorizing the use of their data in the study.

### Participants and settings

The inclusion criteria included women of low socioeconomic status, 18 years of age or older, newly diagnosed with BC, not hitherto subjected to the first cycle of chemotherapy, and procession of a formal approval for participation in the study by the treating physician. The exclusion criteria included lack of response to assessments and withdrawal from participation in the research. Accordingly, a total of 55 women diagnosed with BC and qualifying the inclusion criteria were included for analysis in the present study.

### Clinical data

The enrolled subjects underwent surgical resection or ultrasound-guided core needle biopsy. Analysis of clinicopathological findings by the treating cancer physician confirmed the final diagnosis of BC.

### Cancer staging

Evaluation of the enrolled subjects included complete physical examination, chest X-ray, bilateral mammography, and ultrasound of the breasts, axilla, cervical region, and abdomen prior to surgical resection and core needle biopsy. The TNM stage was assessed according to the 8^th^ edition of the AJCC^(4)^ staging manual.^(9)^

### Pathology analysis

Histological tumor grade was assigned to stained paraffin-embedded BC blocks (n=55) based on the Nottingham Grading System (NGS). The NGS is a modification of the Scarff-Bloom-Richardson (SBR) grading system that grades the degree of differentiation of the tumor using a numerical scoring system assigned as 1 (well differentiated), 2 (moderately differentiated), or 3 (poorly differentiated carcinoma).^(10)^

Estrogen responsive (ER), progesterone responsive (PR) and HER2 status were detected by immunohistochemical (IHC) methods. Stained sections with >1% of total tumor cell nuclei positive were interpreted as ER/PR positive. Stained sections were interpreted for HER2 as either negative, ++ as uncertain, or +++ as positive. Tumor sections that were HER-2 positive together with a score of 2+ were further evaluated by fluorescent *in situ* hybridization. Tumor sections were classified as triple negative if they tested negative for ER/PR, and HER2.^(3)^

### Analysis of PAL and SED

The International Physical Activity Questionnaire (IPAQ) was used to analyze PAL and SED. The IPAQ assessed the frequency, intensity, and duration of physical activity and accordingly classified the individuals into four categories namely, very active, active, non-active, and sedentary. In addition, the total sitting time during the week and weekends was measured^(11)^ and a weighted average was created to evaluate the average sitting time of the week and weekend (weekday sitting time*5 + weekend day sitting time*2, divided by seven days of the week). In this study, the cutoff scores used for PAL were as follows: 1) Active: individuals who perform the following physical activity recommendations; a) Vigorous: ≥5 days/week and ≥30 minutes per session and/or b) Vigorous: ≥3 days/week and ≥20 minutes per session + Moderate physical activity (PA); c) Walking: ≥5 days/week and ≥30 minutes per session; observing the following recommendations for PA; d) Vigorous: ≥3 days/week and ≥20 minutes per session and/or e) Moderate or Walking: ≥5 days/week and ≥30 minutes per session and/or any activity accumulated ≥5 days/week and ≥150 minutes/week (walking + moderate + vigorous); 2) Sedentary: individuals who perform physical activity, but insufficient to be classified as active because they do not comply with the recommendations regarding frequency or duration: a) Frequency: 5 days/week or b) Duration: 11-149 min/week or individuals who stopped exercising to perform continuous physical activity for at least 10 minutes during the week.

In addition, data related to demographic and clinical characteristics of patients such as age, body mass index (BMI; healthy: ≤25Kg/m^2^ and obese: >25Kg/m^2^), marital status, menarche, menopause, number of children, duration of breastfeeding, family history of BC, presence of comorbidities, incidence of metastasis, and existence of lymph nodes were obtained from the medical records.

### Data analysis

The obtained data was evaluated to compare PAL *versus* SED based to their association with staging and histological tumor grade in women with BC.

Statistical analyzes were performed using the (SPSS) for Windows version 24.0 (IBM Corp, Armonk, NY, USA). For categorical variables, participant characteristics were represented as frequencies and percentage, while continuous variables were represented as mean and standard deviations (SDs). The Shapiro-Wilk test was used for analyzing data normality and the χ^2^ test for the association of categorical variables. The Student’s *t*-test and Mann-Whitney test were used for the comparative analysis of numerical variables based on the normality of the data and analysis of variance (ANOVA) for the number of groups. A value of p<0.05 was considered statistically significant.

## RESULTS

The demographic and clinical data of the enrolled study participants are summarized in tables 1 and 2. The study included a total of 55 women diagnosed with BC, of whom, 81.80% were adults (mean age 49.00±10.99 years), 74.50% were obese (mean BMI 28.04±6.97Kg/m^2^), 67.30% did not report any kind of comorbidities, and 50.90% had a family history of cancer. Analysis of clinical parameters presented, 58.20% of the subjects with tumor cells in the lymph nodes, 25.50% with metastasis, 20.00% at a tumor stage of 3, 49.10% with a histological tumor grade of 3, 70.90% were hormone responsive, and 20.00% had triple negative subtype of invasive BC. Analysis of PAL and SED revealed, 54.50% of the subjects to be active with an average sitting time of 4.26±2.35 hours during the week, 4.53±2.28 hours during the weekend, and the average time spent sitting week + weekend was 4.39±2.06 hours.

**Table 1:** Demographic and clinical profile of the enrolled breast cancer patients

Variable	n (%)
Age group	
<60 years	45 (81.80)
≥60 years	10 (18.20)
Marital status	
Single	26 (47.30)
Married	29 (52.70)
BMI	
<25 Kg/m^2^	14 (25.50)
≥25 Kg/m^2^	41 (74.50)
Comorbidities	
No	37 (67,30)
Yes	18 (32.70)
Physical activity level	
Active	30 (54.50)
Sedentary	25 (45.50)
Menopause	
No	33 (60.00)
Yes	22 (40.00)
History of cancer	
No	27 (49.10)
Yes	28 (50.90)
Metastasis	
No	41 (74.50)
Yes	14 (25.50)
Tumor stage	
1	21 (38.20)
2	23 (41.80)
3	11 (20.00)
Tumor grade	
2	28 (50.90)
3	27 (49.10)
Hormone responsive	
No	16 (29.10)
Yes	39 (70.90)
ER	
No	13 (23.60)
Yes	42 (76.40)
PR	
No	22 (40.00)
Yes	33 (60.00)
HER2	
Negative	40 (72.70)
Positive	15 (27.30)
Triple negative	
No	44 (80.00)
Yes	11 (20.00)
Lymph nodes	
No	23 (41.80)
Yes	32 (58.20)
Total	55 (100.00)

n: number of patients; BMI: body mass index; Kg/m^2^: kilograms/meter square; ER: estrogen receptor; PR: progesterone receptor; HER2: human epidermal growth factor receptor type 2.

**Table 2:** Demographic and clinical profile of women diagnosed with breast cancer

Variable	Mean±SD	Median	Minimum	Maximum	n
Age	49.00±10.99	48	26	75	55
BMI (Kg/m^2^)	28.04±6.97	27.85	2.43	54.86	55
Menarche (years)	12.53±2.32	13	0	17	55
Menopause (years)	48.41±4.97	48	38	56	22
Number of children	1.89±1.18	2	0	5	55
Breastfed (months)	12.78±13.97	7	0	48	55
Time sitting in the week (hours)	4.26±2.35	4	1	10	55
Weekend sitting time (hours)	4.53±2.28	4	0,5	10	55
Mean sitting time per week [week + weekend] (hours)	4.39±2.06	4	1.25	9	55

n: number of patients; SD: standard deviation; BMI: body mass index; Kg/m^2^: kilograms/meter square.

Association between PAL, demographic profile, and clinical characteristics of the enrolled subjects are summarized in table 3. Physical activity levels was not significantly associated with the tumor stage (p=0.26) and histological tumor grade (p=0.07). However, PAL was found to significantly influence the response to hormone (p<0.05) and was associated with the expression of HER2 (p<0.05).

**Table 3:** Association between physical activity levels, demographic profile, and clinical characteristics of the enrolled breast cancer patients

Variable	Physical activity level		Total n (%)	p value

	Active n (%)	Sedentary n (%)		
Age group				0.74
<60 years	24 (80.00)	21 (84.00)	45 (81.80)	
≥60 years	6 (20.00)	4 (16.00)	10 (18.20)	
Marital status				0.92
Single	14 (46.70)	12 (48.00)	26 (47.30)	
Married	16 (53.30)	13 (52.00)	29 (52.70)	
BMI				0.82
<25 Kg/m^2^	8 (26.70)	6 (24.00)	14 (25.50)	
≥25 Kg/m^2^	22 (73.30)	19 (76.00)	41 (74.50)	
Comorbidities				0.64
No	21 (70.00)	16 (64.00)	37 (67.30)	
Yes	9 (30.00)	9 (36.00)	18 (32.70)	
Metastasis				0.14
No	20 (66.70)	21 (84.00)	41 (74.50)	
Yes	10 (33.30)	4 (16.00)	14 (25.50)	
History of cancer				0.22
No	17 (56.70)	10 (40.00)	27 (49.10)	
Yes	13 (43.30)	15 (60.0)	28 (50.90)	
Hormone responsive				0.048*
No	12 (40.00)	4 (16.00)	16 (29.10)	
Yes	18 (60.00)	21 (84.00)	39 (70.90)	
RE				0.06
No	10 (33.30)	3 (12.00)	13 (23.60)	
Yes	20 (66.70)	22 (88.00)	42 (76.40)	
PR				0.10
No	15 (50.00)	7 (28.00)	22 (40.00)	
Yes	15 (50.00)	18 (72.00)	33 (60.00)	
HER2				0.02*
Negative	18 (60.00)	22 (88.00)	40 (72.70)	
Positive	12 (40.00)	3 (12.00)	15 (27.30)	
Triple negative				0.18
No	22 (73.30)	22 (88.00)	44 (80.00)	
Yes	8 (26.70)	3 (12.00)	11 (20.00)	
Lymph nodes				0.40
No	11 (36.70)	12 (48.00)	23 (41.80)	
Yes	19 (63.30)	13 (52.00)	32 (58.20)	
Tumor stage				0.26
1	9 (30.00)	12 (48.00)	21 (38.20)	
2	13 (43.30)	10 (40.00)	23 (41.80)	
3	8 (26.70)	3 (12.00)	11 (20.00)	
Tumor grade				0.07
2	12 (40.00)	16 (64.00)	28 (50.90)	
3	18 (60.00)	9 (36.00)	27 (49.10)	
Total	30 (100)	25 (100)	55 (100)	

* χ^2^ test, significance level ≤5%.

n: number; BMI: body mass index; Kg/m^2^: kilograms/meter square; ER: estrogen receptor; PR: progesterone receptor; HER2: human epidermal growth factor receptor type 2.

Comparative analysis of SED, demographic profile, and clinical characteristics of the enrolled subjects are summarized in table 4. Sedentary behavior significantly influenced the histological tumor grade in relation to the mean time spent sitting during the weekend (p<0.05). However, SED did not influence the tumor stage (p=0.32, p=0.60, and p=0.59 in relation to the average sitting time during the week, weekend, and the week + weekend, respectively).

**Table 4:** Comparative analysis of sedentary behavior, demographic profile, and clinical characteristics of the enrolled breast cancer patients

Variable	n	MTSS (hours)		MTSF (hours)		MTST (hours)	
		
		Mean±SD	p value	Mean±SD	p value	Mean±SD	p value
Age group							
<60 years	45	4.34±2.32	0.55	4.52±2.32	0.97 4.20±1.95	4.43±2.11	0.75
≥60 years	10	3.85±2.56		4.55±2.19			
Marital status							
Single	26	3.94±2.05	0.36	4.46±2.05	0.84 4.56±2.34	4.20±1.73	0.53
Married	29	4.53±2.59		4.59±2.50			
Menopause							
No	33	4.67±2.35	0.11	4.92±2.24	0.12 3.78±1.90	4.80±2.10	0.08
Yes	22	3.64±2.25		3.93±2.26			
BMI (kg/m^2^)							
<25 Kg/m^2^	14	3.61±1.86	0.18	3.71±2.10	0.12 4.64±2.13	3.66±1.70	0.13
≥25 Kg/m^2^	41	4.48±2.47		4.81±2.30			
Comorbidities							
No	37	4.16±2.30	0.68	4.26±2.26	0.21 4.76±2.16	4.21±2.02	0.36
Yes	18	4.44±2.50		5.08±2.28			
Cancer of history							
No	27	3.82±2.02	0.18	4.24±2.29	0.37 4.74±2.23	4.03±1.84	0.20
Yes	28	4.68±2.59		4.80±2.27			
Metastasis							
No	41	4.32±2.44	0.74	4.51±2.46	0.93 4.32±1.60	4.41±2.22	0.89
Yes	14	4.07±2.13		4.57±1.74			
Responsive hormone							
No	16	3.50±1.79	0.08	3.63±2.09	0.06 4.73±2.15	3.56±1.59	0.06
Yes	39	4.56±2.50		4.90±2.28			
Triple negative							
No	44	4.30±2.37	0.80	4.53±2.27	0.97 4.30±2.22	4.41±2.05	0.87
Yes	11	4.09±2.39		4.50±2.42			
Lymph nodes							
No	23	4.04±2.36	0.58	4.91±2.41	0.29 4.33±1.97	4.48±2.23	0.79
Yes	32	4.41±2.37		4.25±2.18			
Tumor stage							
1	21	4.05±2.55	0.32	4.91±2.58	0.60 4.59±2.07 3.82±1.19	4.48±2.42	0.59
2	23	4.78±2.32		4.39±2.33			
3	11	3.55±1.92		4.09±1.51			
Tumor grade							
2	28	4.07±2.55	0.56	3.95±2.16	0.048* 4.79±1.98	4.01±2.11	0.16
3	27	4.44±2.15		5.13±2.29			

* Student *t*-test, significance level ≤5%.

n: number of patients: MTSS: mean sitting time during the week; MTSF: mean sitting time during the weekend; MTST: mean sitting time throughout the week (week + weekend); SD: standard deviation; BMI : body mass index; Kg/m^2^: kilograms/meter square.

## DISCUSSION

The major findings of the present study indicate a significant influence of PAL with tumor responsiveness, HER2 expression, and histological tumor grade in women diagnosed with BC. As per the descriptive analysis, of the total subjects enrolled in the study, 84% were hormone-responsive and 76% were obese. Earlier studies have indicated obesity and associated physical inactivity as a cause of enhanced insulin resistance,^(12,13)^ impaired glucose consumption and tolerance,^(14)^ inactivation of insulin receptor, and enhanced expression of insulin-like growth factor receptors (IGFR)^(15)^ and human epidermal growth factor receptor (HER2).^(16)^

In the present study, 88% of sedentary subjects were HER2 negative. Epidermal growth factor (EGF) receptors are commonly dysregulated in human cancers.^(17)^ Accordingly, enhanced expression of HER2 serves as a biomarker and prognostic predictor for BC.^(18)^ A previous study considering sex, age, and BMI in non-cancerous individuals suggested an association of enhanced HER2 expression with insulin resistance, type 2 *diabetes mellitus*, and IGFR.^(19)^ Data from our study revealed 16% of sedentary women to be elderly, 76% to be obese, and 64% with no comorbidities, all of which serve as important factors for enhanced HER2 expression. Majority of the subjects diagnosed with BC were at an early stage of the disease, wherein 74.5% were non-metastatic, 50.9% had a histological tumor grade of 2, and 49.10% had a histological tumor grade of 3. In addition, 20% of the subjects diagnosed with BC were at a tumor stage of 3. The study was limited in analyzing the characteristics of enrolled subjects with a histological tumor grade of 1 and at a tumor stage of 4, considering the advanced stage of the disease. However, the present study design might influence this finding, since the assessment was performed at the first diagnosis of BC signifying the early stage of tumor development.

SED and PAL plays an important role in increasing the risk of BC.^(20-22)^ However, the role of SED in the prognosis of BC remains to be elucidated. The present study indicates significant difference in the histological tumor grade in relation to variations in SED. A higher mean time spent sitting during the weekend was observed in BC subjects with a histological tumor grade of 3 than those with a histological tumor grade 2 (5.13±2.29 hours and 3.9±-2.16 hours, respectively, p<0.05). However, the behavior could not be verified in relation to the average sitting time during the week and the average sitting time throughout the week (week + weekend). The findings might have been influenced by the sample characteristic, wherein 54.4% of the subjects were active, as majority of the subjects had an active occupational activity (daily, housekeeper, cleaning assistant, hairdresser). In addition, although validated in the literature, the presented results are influenced by the questionnaires used to assess PAL.

The current study reveals novel and interesting results in deciphering the relationship between PAL and anatomopathological variables of BC. However the study presents certain limitations. The small sample size limits the understanding of adjusted variables in the outcome. An important highlight is the possibility of simultaneous occurrence of meeting the global recommendations of physical activity and having elevated sitting time in the same participant. Thus, mutually adjusted analysis would help to clarify independent associations between physical activity variables and BC. The cross-sectional design of the study limits the understanding of causal relationship between indicators of PAL and SED with BC. However, the present study serves as a parameter for the design of future studies to better understand the relationship between the anatomopathological variables of BC and PAL.

## CONCLUSION

The present cross-sectional study design enrolled women diagnosed with breast cancer and revealed tumor staging and histological tumor grade to be independent of physical activity levels. However, physical activity levels significantly influenced the hormonal response and HER2 expression. In addition, significant association was detected between sedentary behavior (time spent sitting during the weekend) and the histological tumor grade.
